# The Relationships between Morphological Characteristics and Foraging Behavior in Four Selected Species of Shorebirds and Water Birds Utilizing Tropical Mudflats

**DOI:** 10.1155/2015/105296

**Published:** 2015-08-09

**Authors:** Nor Atiqah Norazlimi, Rosli Ramli

**Affiliations:** Institute of Biological Sciences, Faculty of Science, University of Malaya, 50603 Kuala Lumpur, Malaysia

## Abstract

A study was conducted to investigate the relationship between the physical morphology of shorebirds and water birds (i.e., Lesser adjutant (*Leptoptilos javanicus*), Common redshank (*Tringa totanus*), Whimbrel (*Numenius phaeopus*), and Little heron (*Butorides striata*)) and their foraging behavior in the mudflats area of Selangor, Peninsular Malaysia, from August 2013 to July 2014 by using direct observation techniques (using binoculars and a video recorder). The actively foraging bird species were watched, and their foraging activities were recorded for at least 30 seconds for up to a maximum of five minutes. A Spearman Rank Correlation highlighted a significant relationship between bill size and foraging time (*R* = 0.443, *p* < 0.05), bill size and prey size (*R* = −0.052, *p* < 0.05), bill size and probing depth (*R* = 0.42, *p* = 0.003), and leg length and water/mud depth (*R* = 0.706, *p* < 0.005). A Kruskal-Wallis Analysis showed a significant difference between average estimates of real probing depth of the birds (mm) and species (*H* = 15.96, *p* = 0.0012). Three foraging techniques were recorded: pause-travel, visual-feeding, and tactile-hunting. Thus, morphological characteristics of bird do influence their foraging behavior and strategies used when foraging.

## 1. Introduction

Shorebirds are a highly mobile group of animals and have sophisticated site-sampling processes that operate on larger spatial scales than most other animals [1]. Shorebirds generally forage during low tide and can be observed on beaches, intertidal mudflats, freshwater and brackish wetlands, farmland, and salt marshes [2]. Meanwhile, water birds refer to the bird species that entirely depend on wetlands for a variety of activities such as foraging, nesting, loafing, and moulting [3]. Both shorebirds and water birds are the important components of estuarine mudflats.

Estuarine mudflats are very important for many shorebird populations during winter and migration, many species of which feed almost exclusively on intertidal benthic invertebrates at low tide [4]. Besides that, mudflats in estuaries are also vital feeding habitats for resident bird populations [5]. In tropical regions, the biodiversity of benthic macrofauna on intertidal mudflats is much higher; macrofauna are produced ten times faster here than in temperate intertidal habitats [6, 7]. During migratory seasons, foraging is the most important activity for shorebirds utilizing the mudflats area, as it allows them to survive and ensures their safe arrival at the breeding ground. The foraging ecology is often characterized by food selection, habitat preference, and prey capturing tactics or behavior employed by avian species in a particular habitat [8, 9].

The morphology of a bird is considered an important factor in restricting the ranges of foraging maneuvers it can perform [10, 11]. Bill length and shape have important implications on foraging behavior [12–15], microhabitat selection [16], and choice of diet [15, 17–19]. Longer bills are associated with probing depth and plunging or sweeping bill movements in the water, while shorter bills are associated with routing and pecking at the substrate surface [14]. Furthermore, the foraging depths are also correlated with culmen and tarsus lengths [20]. The bill's shape (either straight or curved) influences the foraging techniques used by* Calidris mauri* (Western sandpipers) [15]. Pecking or feeding on epifaunal invertebrates is associated with a straight bill, while probing or feeding on infaunal prey is facilitated by bill curvature. In terms of foraging strategies, the functional requirement of a tactile foraging strategy is a high penetration capacity, which is then influenced by the morphological characters of a bird's bill [13]. The general morphological requirements necessitate that the bill be long and narrow but not very slender, and the penetrating portion should be flattened either vertically or horizontally. Time spent feeding also varies with respect to the size of the bird [21]. Larger birds spent less time foraging than smaller birds by eating larger and more profitable prey.

The majority of the studies on the foraging behavior of shorebirds and water birds were conducted in temperate climate areas. The feeding ecology of shorebird and water birds species in tropical countries, especially Malaysia, is poorly understood. A previous study [28] focused on the correlation between bird density and prey density, whereas one study [29] focused on birds' habitat utilization. To date, no detailed information has been obtained on the correlation between the morphological characteristics of birds and their feeding ecology in Malaysia. Therefore, this study aims to determine the significant relationships between morphological characters and foraging behavior adapted by shorebird and water birds species utilizing the mudflats area of Jeram Beach and Remis Beach, Selangor, Peninsular Malaysia.

## 2. Materials and Methods

### 2.1. Study Area

**Figure 1:**
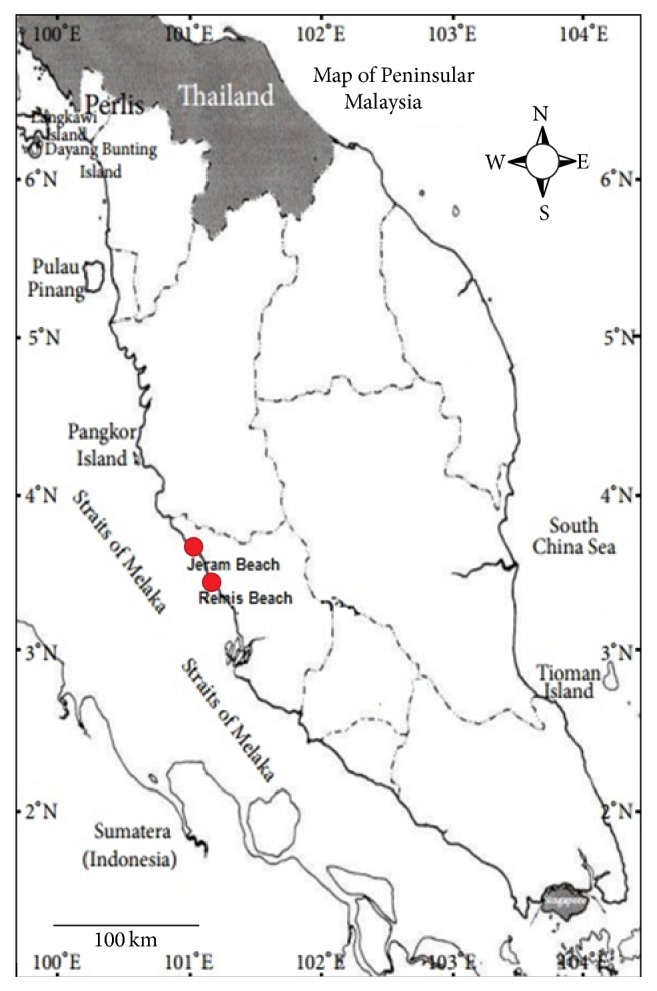
The Coastal Mudflats of Jeram and Remis Beaches, Selangor, West Coast of Peninsular Malaysia.

The Jeram and Remis Beaches are located on the Selangor Coast on the West Coast of Peninsular Malaysia (3°13′27′′N, 101°18′13′′E) (Figure 1) where semidiurnal tides prevail. In Jeram Beach, the flat was fringed by a mangrove stand of stunted* Avicennia alba* Blume and few scattered* Sonneratia* spp. [30]. The distance between Jeram Beach and Remis Beach is approximately 2 km. The selected study areas comprise approximately 55 ha of intertidal mudflats, that is, 27 ha on Jeram Beach and 28 ha on Remis Beach. The selection of these sites was based on past shorebird counts reported by Wetland Internationals from 1999 to 2004 [31], which shows that these areas were previously known to be important stopover sites for shorebirds.

### 2.2. Foraging Behavior

The foraging behavior of birds was studied from August 2013 to July 2014 using direct observation techniques. Selected focal birds were observed using binoculars (12 × 42 magnifications), stopwatches, and video recorders. The selected focal bird must be actively foraging (each individual was observed until they were done foraging, i.e., starting from the time the bird began actively searching for prey until the prey was completely swallowed); if the bird left within 30 seconds, it was eliminated from the study [32]. The focal observations were recorded for at least 30 seconds for up to a maximum of five minutes. The data recorded from the different sites and months were pooled to increase replications [33] so that the data was strong enough to be analyzed. The focal observations were done only during low tide period (i.e., during ebbing tide, low tide peak, and rising tide) so that birds of all sizes (either with longer or shorter legs) can use the mudflats area for foraging at the same time. The observations were conducted during four-interval period (i.e. 0800–1000 hours, 1000–1200 hours, 1400–1600 hours, and 1600–1800 hours). Since the sites were situated nearest to each other (i.e., 2 km apart), the differentiation of habitat characteristics was not obvious. First, individual focal birds were selected from a flock. Once a bird was chosen, the next bird selected for observation must be located at least 10 meters away from the previously observed bird. This was done to avoid multiple observations of the same individual. Four species of shorebirds and water birds (Lesser adjutant (*Leptoptilos javanicus*), Common redshank (*Tringa totanus*), Little heron (*Butorides striata*), and Whimbrel (*Numenius phaeopus*)) were chosen for this study due to their size differences and foraging techniques and because they are easily distinguished from one another. The data, such as estimated probing depth, time spent foraging (total searching and handling time), foraging techniques, prey type, estimated prey size, and estimated water or mud depth in which they forage, were gathered. Foraging techniques used by bird were divided into three categories: (1) tactile-hunting species techniques, where birds forage as they walk, probing continuously with the bill into the substrate [34, 35]; (2) visual-feeding techniques, where they forage in a continuous fashion, pecking at items seen on the substrate surface [36] and; (3) pause-travel species techniques, where they mainly forage by scanning the area in front of them and pecking at the substrate surface when prey is detected in a stop-run-stop fashion [37, 38]. Five prey items were observed to be the main diet choice for shorebirds: fish, bivalves, worms, crabs, and unknown (aquatic insects or invertebrate fauna). Preys were classified as “unknown” when they could not be clearly seen. The size and number of prey items taken by these birds were estimated and recorded. Bill length was used to estimate probing depth and prey size, while leg length was used to estimate water or mud depth based on the maximum percentage (%) of the leg that was immersed in the water or mud. The length of the leg of selected birds was estimated by doubling the length of the tarsus. Similarly, the probing depth was estimated based on the maximum percentage of bill length inserted into the mud or water. Meanwhile, prey size was estimated based on the percentage of the prey items inserted into the bill.  Table 1 summarizes the measurement of the bill size and tarsus length of the bird species based on previous studies. Below are the formulae used to illustrate the estimation of probing depth and water or mud depths:

(1) Water/mud  depthmm=Percentage  of  estimated  leg  immersed  in  the  water  or  mud100%×leg  length,Probing  depth  mm=Percentage  of  estimated  bill  inserted  into  the  mud  or  water100%×bill  size,Prey  size  mm=Percentage  of  estimated  prey  in  bill100%×bill  size.

### 2.3. Data Analysis

Statistical software [39] was used to analyze all data. In preparation for statistical testing, all data sets were tested with Shapiro Wilk's *W* test and Anderson's Darling test for normality. In all cases, *α* = 0.05 was used. A total of 205 focal observations were recorded for Common redshank, 75 observations for Lesser adjutant, 53 observations for Little heron, and 38 observations for Whimbrel (Table 2). Due to differences in number of focal observations recorded, all data taken were divided into 12 months (i.e., from August 2013 until July 2014) to obtain the average or mean of each data. A Spearman Rank Correlation Analysis was used to determine the correlation between the bill size of the bird and the time spent foraging [40]; the bill size of the bird and the estimated prey size; the bill size of the bird and probing depth; and the leg length of the bird and water or mud depth. The nonparametric Kruskal-Wallis Test was used to study the relationships between bird species and probing depth (mm). Moreover, a one-way ANOVA was used to determine the differences in time spent foraging and different foraging techniques. All the requisites of data reliability have been followed [41]. The statistical test used was based on [42].

## 3. Results

Our results show that time of the day and tidal conditions do not influence the use of habitat for foraging by shorebird and water bird species utilizing the areas of the study. One-way ANOVA analysis was conducted between four intervals periods (i.e., 0800–1000, 1000–1200, 1400–1600, and 1600–1800 hours) and the results show no significant difference in the time of birds species that used the habitat for foraging during the intervals (*F* = 1.86, *p* = 0.190). Similarly, no significant differences were found during different low tide period (i.e., ebbing tide, low tide peak, and rising tide) (*F* = 1.62, *p* = 0.251). Furthermore, by using Friedman's Two-Way Analysis of Variance by Ranks Test, the results show no significant difference for searching time of preys between species (*F* = 4.92, *p* = 0.178). On the contrary, the handling times were significantly different between the bird species (*F* = 19.49, *p* < 0.05). Further analysis by using Pairwise Comparisons Test highlighted that the differences occurred between Lesser adjutant and Whimbrel (*z* = −1.7, *p* = 0.019) and also between Common redshank and Little heron (*z* = 1.6, *p* = 0.034). The differences in handling time were found to influence the total time spent for foraging between the birds species (*F* = 13.3, *p* = 0.004). Pairwise Comparisons Test proved that the significant difference occurred between Little heron and Lesser adjutant (*z* = −1.667, *p* = 0.009) and between Lesser adjutant and Common redshank (*z* = 1.583, *p* = 0.016). In this study, we found that the time spent foraging, prey size, and probing depth differed with respect to the bill size of the shorebird and water bird species. Moreover, the water or the mud depth where the bird stood while foraging was also influenced by the length of the leg of the bird species. A Spearman Rank Correlation shows a significant relationship between bill size and time spent foraging (*R* = 0.443, *p* < 0.05); the bill sizes and the estimated prey size obtained while foraging (*R* = −0.052, *p* < 0.05); the bill sizes and the probing depth applied while foraging (*R* = 0.42, *p* = 0.003); and the length of the leg and the water or mud depth in the feeding area (*R* = 0.706, *p* < 0.005) (Table 3).

We also found that probing depth varied between species. A Kruskal-Wallis Analysis shows a significant difference between average estimates of real probing depth (mm) and species (*H* = 15.96, *p* = 0.0012). Bonferroni's post hoc test, with a correction of *α* = 0.05, was calculated, and the results showed that the significant difference exists only between the Little heron and the Lesser adjutant (*z* = 3.97, *p* = 0.001).

In terms of diet choice, five prey items were observed to be the main diet choice for shorebirds and water birds: fish, bivalves, worms, crabs, and unknown (Table 4). Based on observations, the bivalve was the most preferred option among bird species, which accounted for 34% of the diet, followed by fish, 29%, unknown, 18%, crab, 12%, and worm, 7%.

Table 5 shows foraging techniques practiced by shorebird and water bird species. Little herons only practiced pause-travel techniques, while Lesser adjutants and Common redshanks used all techniques while foraging. However, the most preferred feeding technique used by Lesser adjutants and Common redshanks was the tactile-hunting feeding technique. In contrast, the Whimbrel engaged in both tactile-hunting and visual-feeding techniques, but not pause-travel techniques. No significant differences were found between time spent foraging and different feeding techniques (*F* = 0.26, *p* = 0.778).

## 4. Discussion

We found that time of the day and tidal conditions do not influence the foraging behavior of shorebird and water bird species utilizing the areas of the study. Similar results were found by [43] through her study of tidal flats, which showed that time of day did not significantly affect variability in shorebird abundance and use of habitat. Previous study by [44] found that the abundance of the birds reached peak between 1.5 and 2.5 hours after low tide which suggests that the availability of food is the greatest during this period. However, surprisingly, our results were in contrast with this study. Any subtle differences in the bird's morphological traits, such as the length of the wing, tarsus, or toes, could result in different foraging maneuvers [45]. This study revealed that the differences in bill size and leg length of the shorebird and water bird species influence the time spent foraging, the size of the prey, the probing depth, and the preference of habitat while foraging. The longer the bill, the more time spent foraging, the larger the prey, and the deeper the area they preferred to forage. Based on our observations, Lesser adjutants and Little herons had longer bills and their diets mainly comprised of larger prey items such as fish. Larger prey required longer swallowing and digesting times, allowing birds to spend more time foraging. Increasing the time spent handling the prey resulted in an increase in the time spent foraging. On the contrary, birds with shorter bills (the Common redshank) were observed to feed on bivalves more frequently. Smaller prey reduced handling time and, thus, reduced time spent foraging. Similar results have been reported [46], which show that birds with longer bills generally feed on larger prey than birds with shorter bills. Probing depth was hypothesized to increase as the length of the bird's bill increased. Birds with longer bills (Lesser adjutants) were observed to probe in deeper mud and higher water as compared to other bird species. A study of the differences in bill sizes of male and female Western Sandpipers (*Calidris mauri*) [47] found that females, who have longer bills, foraged in sites with a higher water content than males did, where the probing technique may be more effective [16]. Although birds with longer bills probed deeper than shorter billed birds, the percentage of which the bill inserted into the mud or water while foraging was differed. Shorter billed birds tended to insert the majority or all of their bills into the mud or water while foraging. Usually, the Common redshank inserted the majority of its bill into the mud while foraging, whereas the Lesser adjutant, Whimbrel, and Little heron only inserted their bills halfway or less while foraging. Deeper probing resulted in a more profitable prey item. The size of the prey increased with respect to burrowing depth. A previous study [48] found that a larger worm species (*Nereis diversicolor*), which is longer than 10 cm, was usually found at a depth of 10 to 14 cm.

The longer the leg, the deeper the mud or water depth in which the birds stood while foraging. This study revealed that the Lesser adjutant tended to forage in the deeper mud and areas close to the water's edge. Meanwhile, the Common redshank was commonly found utilizing the area closest to the beach, which was shallower and drier compared to the area closest to the water's edge. Similar results [49] show that shorebirds with shorter legs and tarsi (i.e.,* Calidris minutilla* (Least Sandpiper),* Calidris mauri* (Western Sandpiper),* Limnodromus* spp. (Dowitcher), and* Calidris alpina* (Dunlin)) were constrained to use mudflats or shallow water zones along the wetland's edge. Leg length was positively correlated with water depth in which shorebirds foraged [20]. Other data [50] also revealed an increase in the range of depths used by larger shorebird species, which wade in deeper habitats. Foraging close to the water's edge might be advantageous because of increased penetrability and prey activity [51]. Therefore, drier substrates and more structurally complex microhabitats may be favored by birds with shorter bills [52, 53].

Our results show a significant difference between average estimates of probing depth and species. The differences in probing depth exist only between Little herons and Lesser adjutants. This may be due to differences in their bill sizes. Lesser adjutants have longer bills than Little herons. Birds with longer bills will benefit by probing deeper into the mud. The differences in habitat use exist in sandpipers due to variations in bill length [54]; that is, longer billed individuals foraged in muddier habitats than did shorter billed individuals.

The foraging techniques engaged in while foraging also differed between species. Tactile hunting was the most dominant technique used by the Lesser adjutant, Whimbrel, and Common redshank, whereas the pause-travel technique was the only technique used by the Little heron. The different types of feeding techniques are likely to influence the vigilance patterns of shorebird species. Pause-travel species can be more vigilant with their heads up, scanning the environment; when they locate a prey item, they run to catch it [55]. We assume that tactile-hunting techniques increase the chances of successful foraging, since much of the bird's time is concentrated on searching for food, compared to pause-travel techniques, in which the bird spends much of its time being more vigilant than foraging. Moreover, shorter billed birds were restricted to a certain mud depth or water level compared to the longer billed bird. Therefore, tactile-hunting techniques were observed to be the most profitable, since the bird using this technique will probe as deep as possible to obtain more profitable prey, which burrow deep into the mud. Our study suggests that time spent foraging did not differ between foraging techniques. However, different results have been shown [56], where Plovers, which exhibit visual foraging techniques, spend less time feeding than Sandpipers, which exhibit tactile or continuous hunting techniques. Furthermore, the pause-travel species was frequently observed foraging alone, whereas tactile and visual feeding species usually foraged in intraspecies or interspecies flocks. Foraging in groups is beneficial because it reduces the risk of predation and, thus, reduces the cost of vigilance [32]. For conclusion, the morphologies of birds play an important role in determining foraging behaviors. Species with different foraging strategies will acquire food resources from different habitats and may be able to avoid interspecies competition. Thus, sufficient energy and nutrients can be replenished to enhance the survival of bird species in the area.

## Figures and Tables

**Table 1:** Measurements of bill size and tarsus length based on previous studies.

Species	Literature	Bill length	Average bill length	Tarsus length	Average tarsus length
		(mm)	(mm)	(mm)	(mm)
Lesser adjutant	[22]	266.7	266.7	228.6	228.6

Common redshank	[23, 24]	43.7	42.8	51.6	49.6
		41.8		47.6	

Little heron	[25]	75.0	75.0	49.0	49.0

Whimbrel	[26, 27]	87.2	84.6	55.9	59.9
		82.0		63.8	

**Table 2:** Summary of frequency of individual of shorebird and water bird species observed (*n*) from August 2013 until July 2014.

Months	Species (*n*)			
	Lesser adjutant	Common redshank	Little heron	Whimbrel
August	6	4	8	4
September	10	20	2	3
October	11	42	2	2
November	3	23	4	2
December	7	33	7	4
January	9	19	5	4
February	5	17	3	4
March	5	17	5	3
April	5	11	5	3
May	6	10	5	3
June	4	5	4	3
July	4	4	3	3

	75	205	53	38

**Table 3:** Summary of bill size, average estimated probing depth, length of leg, average estimated water/mud depth per year, and average time spent foraging by shorebirds and water birds.

Species	Bill size (mm)	Estimated probing/ foraging depth (mm)	Length of the leg (mm)	Estimated water/ mud depth (mm)	Time spent foraging (s)
Little heron	75	24	98	27	1,130
Lesser adjutant	266.7	82	457.2	134	3,186
Whimbrel	84.6	41	119.8	22	2,085
Common redshank	42.8	33	99.2	38	1,280

**Table 4:** Diet choice and abundance chosen by shorebird and water bird species.

Species	Prey type	Number of prey counted
Little heron	Fish	35
	Bivalve	0
	Worm	0
	Crab	0
	Unknown	17

Lesser adjutant	Fish	51
	Bivalve	7
	Worm	2
	Crab	2
	Unknown	15

Whimbrel	Fish	8
	Bivalve	17
	Worm	1
	Crab	7
	Unknown	0

Common redshank	Fish	13
	Bivalve	102
	Worm	25
	Crab	34
	Unknown	35

**Table 5:** Sample size (*n*), mean and standard error of time spent foraging, and foraging techniques used by species.

Species	Foraging techniques	Time spent foraging (s)		
		*n*	Mean	Standard error
Little heron	Pause-travel	53	68.62	5.69
	Tactile-hunting	0	0.00	0.00
	Visual-feeding	0	0.00	0.00

Lesser adjutant	Pause-travel	17	134.65	18.50
	Tactile-hunting	56	77.34	8.13
	Visual-feeding	2	24.00	4.00

Whimbrel	Pause-travel	0	0.00	0.00
	Tactile-hunting	33	36.70	2.03
	Visual-feeding	5	120.00	0.00

Common redshank	Pause-travel	2	50.00	0.00
	Tactile-hunting	171	46.09	2.58
	Visual-feeding	32	39.53	2.54
